# Investigation of Porous Carbon Fibers Based on Porous Polyacrylonitrile Fibers Using High-Resolution Methods across Different Scales

**DOI:** 10.1021/acsomega.5c04460

**Published:** 2025-09-23

**Authors:** Iris Kruppke, Mohsen Sadeghi Bogar, Jan Wolf, Paul Bertram, Michael Thomas Müller, Magdalena Eder, Anke Dutschke, Adrian Mikitisin, Pragya Shekhar, Frank Hitzel, Matthias Finger, So Jeong Heo, Bon-Cheol Ku, Jizhen Zhang, Peter A. Lynch, Gregory Pognon, Gabriel Foyer, Robert Seidel-Greiff, Thomas Behnisch, Irina Kuznik, Markus Löffler, Darius Pohl, Bernd Rellinghaus, Maik Gude, Chokri Cherif

**Affiliations:** † 9169 Dresden University of Technology, Institute for Textile Machinery and High Performance Material Technology, Hohe Str. 6, Dresden 01069, Germany; ‡ 9169 Dresden University of Technology, Research Center Carbon Fibers Saxony, Haus D Breitscheidstraße 78, Dresden 01237, Germany; § Dresden University of Technology, Institute of Lightweight Engineering and Polymer Technology, Holbeinstr. 3, Dresden 01307, Germany; ∥ Department of Processing Technology, Leibniz Institut für Polymerforschung Dresden e.V., Hohe Strasse 6, Dresden 01069, Germany; ⊥ Zeiss Research Microscopy Solutions, Carl Zeiss-Str. 22, Oberkochen D-73447, Germany; # Gemeinschaftslabor für Elektronenmikroskopie (GFE), 9165 RWTH Aachen University, Ahornstraße 55, Aachen 52074, Germany; ∇ Center for Ageing, Reliability and Lifetime Prediction for Electrochemical and Power Electronic Systems (CARL), RWTH Aachen University, Campus-Boulevard 89, Aachen 52074, Germany; ○ DoubleFox GmbH, Geysostraße 13, Braunschweig 38106, Germany; ◆ 317006 WITec GmbH (Part of the Oxford Instruments Group), Lise-Meitner-Strasse 6, Ulm 89081, Germany; ¶ Carbon Composite Materials Research Center, Institute of Advanced Composite Materials, 58975 Korea Institute of Science and Technology (KIST), 92, Chudong-ro, Bongdong-eup, Wanju-gun, Jeollabuk-do 55324, South Korea; ⟁ Research Center for Materials Nanoarchitectonics (MANA), 1-1 Namiki, Tsukuba, Ibaraki 305-0044, Japan; △ Institute for Frontier Materials, Deakin University, 75 Pigdons Road, Geelong VIC 3216, Australia; ▲ 84360 THALES Research and Technology, 1 avenue Augustin Fresnel, Palaiseau Cedex F-91767, France; ▽ 28394 Dresden University of Technology, Dresden Center for Nanoanalysis, Dresden 01062, Germany

## Abstract

This work presents a new cross-scale approach to evaluating the carbon fiber structure. This is demonstrated along the manufacturing chain from porous polyacrylonitrile (PAN) precursor fibers to porous carbon fibers (CF), where the particular challenge lies both in maintaining the porous structure throughout all process steps of thermal conversion and in ensuring that the fiber is sufficiently strong for processing in the stabilization and carbonization steps. In order to achieve this, an electron beam treatment was used to cyclize and cross-link the PAN for prestabilization, whereby the cyclization index was determined on the basis of spectroscopic methods and was 54% for the subsequently thermally stabilized fiber. The produced fibers were characterized by classical textile-physical investigations along the process chain, as well as by means of analyses with regard to structural changes and radial distribution of carbon crystallites, pores, and cavities, which were carried out using X-ray techniques, including wide-angle X-ray diffractometry (WAXS) and nanoscale X-ray tomography (XRM), as well as density measurements, and imaging techniques such as scanning electron microscopy (SEM) with *in situ* Raman spectroscopy and *in situ* atomic force microscopy, as well as transmission electron microscopy (TEM). The results indicate that structural changes take place in the radial direction and that a distinct core–sheath structure also forms for porous fibers. The initially determined specific surface area of the porous PAN fiber was 39.95 m^2^/g and decreased to 0.99 m^2^/g for the porous CF, whereby the density of the latter is significantly lower than that of standard CF, at 1.537 g/cm^3^. These findings provide a framework for a deeper understanding of the structure–property relationships along the carbon fiber manufacturing chain in order to develop tailored porous CF for CF-based supercapacitors.

## Graphical abstract

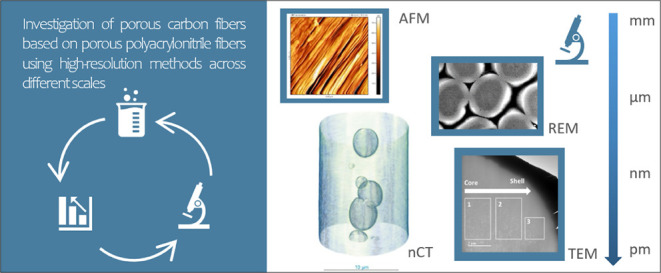

## 1 Introduction

Carbon fibers (CF) have secured their place in the field of structural materials for decades. Proven advantages such as high strength (3–7 GPa) and high modulus (200–500 GPa) combined with very low weight/density (1.7–2 g/m^3^) and high chemical resistance have ensured their use in a wide variety of areas of life and industry.^1–3^ These include original applications such as aerospace, construction,^4^ wind turbines, and energy generation, as well as the automotive,^5^ transportation, and sports industries.^1,6^ Due to the originally very compartmentalized development origins and progression in the military and aerospace industries, the established process steps of carbon fiber production are taken for granted. Here, process steps and parameter windows have been established that are oriented toward the properties of the high-performance fibers to be produced. In the past decade of research and development, a number of research groups have begun to focus on alternative precursor materials, processes^7–16^ as well as alternative energy sources^17,18^ and process optimization.^19–22^ However, in order to understand this in relation to the materials used and the changes in the precursor over the manufacturing process and to understand the complex structural changes and structure–property relationships during the energy-, time-, and cost-intensive conversion into a carbon fiber, various instrumental methods are required to represent these complex relationships.

The production of carbon fibers usually involves the complex process steps of precursor fiber production, mostly polyacrylonitrile (PAN) fibers and stabilization, followed by carbonization. In special cases, graphitization is also carried out. The fiber formation of this is highly dependent on the materials used (precursor) and the subsequent treatment steps. Negative influences during precursor spinning, as well as the composition of the precursor, e.g., modified by copolymers (acrylic acid methyl ester, itaconic acid, methyl methacrylate, or acrylamide) or additives,^3,23^ influence the PAN fiber structure and consequently the CF structure. Even the smallest changes in the process can lead to turbulence in the PAN filament formation in the coagulation bath or changes in the stretching regime so that the filament formation is disturbed and the polymer chains do not combine optimally. This can lead to deviations in fiber’s cross-section geometry (no longer round, but heart- or kidney-shaped), hollow fiber geometry, microvoids, pores, or other defects in the precursor fiber.^24–26^ In addition, such effects or defects that occur are further modified or intensified in subsequent thermally driven process steps. The conversion of the relatively polymer-chain-based structure by cyclization and dehydration reactions results in ring formation, which is further intensified by carbonization. During the conversion to CF, these defects usually remain in the precursor or change into other, carbon (C)-specific stacking (0.3–0.7 nm) and structural defects, such as C-vacancies/C-vacancy clusters (0.2–6 nm).^27^ All heteroatoms are split off, resulting in a turbostratic graphitic structure.^1,3,28^ According to this, undefined structural and macroscopic defects, such as blistering and pores, can arise from gas formation (N2, H2, and O2) during cross-linking reactions in C-structure formation and at high kinetic speeds. These defects are almost impossible to repair^29^ or can only be partially reduced by thermodynamic processes at higher temperatures, which has only a limited positive effect on the mechanical properties of the CF.^30^ In these process steps, also, stretching conditions, temperature profiles, and residence times have a significant influence on the structural transformation mechanisms, the formation of the graphitic fractions, and the turbostratic structure (Figure 1C), as well as the resulting macroscopic properties.^1,26^ These effects are also known for carbon nanotubes (CNT). Here, small C structures, such as CNT, rearrange at higher temperatures into an energetically and thermodynamically optimized C layer structure. Targeted manipulation of the pore structure and size (between 2–5 nm and 40–60 nm) toward hierarchically structured, interconnected pore systems with a high specific inner surface area (2000 m^2^/g) can be used to increase the energy storage capacity of CF.^31^

**Figure 1:**
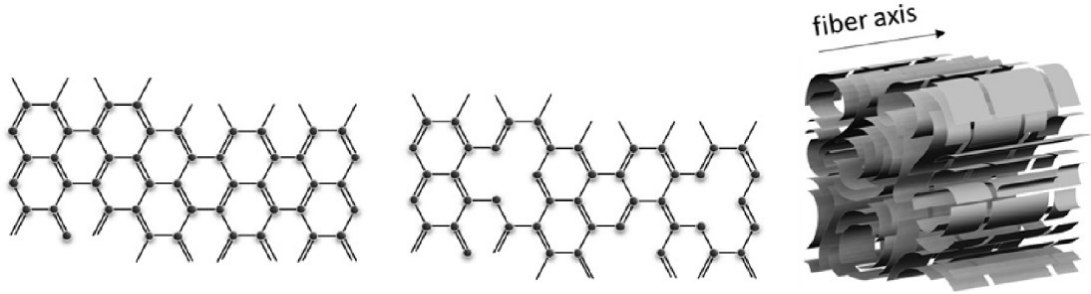
Schematic graphitic structure (A), structural defects and C-vacancies (B), and schematic turbostratic structure along the fiber axis (C).

Carbon fibers do not have an ideal graphene-like structure for manufacturing reasons (Figure 1A), since morphological effects such as cracks and pores, as well as material and structural defects at the atomic and molecular level, such as C-vacancies and misaligned C layers (Figure 1B), play a dominant role due to precursor material, stacking faults, and disclinations.^3^ These defects can, for example, lead to a significant reduction in stiffness and strength as well as affect electrical or thermal conductivity. However, the exact formation mechanisms, interactions, propagation, and their influence on the final properties of carbon fibers must be been fully understood.^25,27,32,33^ From a global perspective, processes occur at the nano-, micro-, and macroscale. Due to the aforementioned, rather closed original development of the carbon fibers, only a limited amount is known about the entire process chain across these scale-crossing process-property relationships and is only relatively well understood for standard qualities.

In general, all classical analytical methods can be used to elucidate these processes and obtain a detailed understanding of the structural transformation mechanisms into carbon fibers. To achieve this goal, the individual analytical methods must be coordinated and their levels and scales of observation must overlap (see Figure 2).

**Figure 2:**
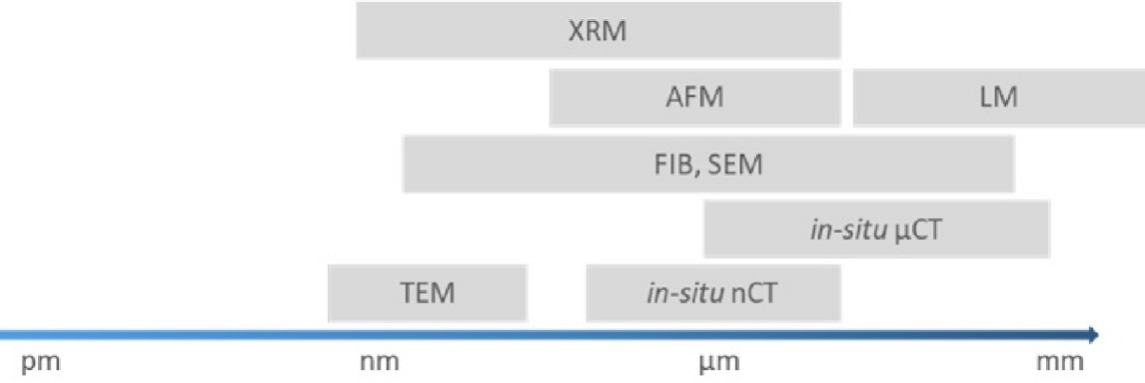
Schematic overview according to the different scales of destructive, nondestructive, surface-related, and radiographic analysis methods.

The aim of this work is to elucidate and present the structure of porous carbon fibers using analytical methods and to relate it to the process steps that have been carried out, on the basis of porous carbon fibers, in a correlative and cross-scale manner. This should provide an initial approach to the complex process­(parameter)–structure–property relationships of CF. Initially, possible investigations during precursor spinning were dispensed with. However, for the sake of completeness, the main parameters and influencing factors that can affect the formation of CF, or pores and defects in the spinning process, are to be mentioned: the molecular weight and distribution of the polymer/copolymer, the type of the polymer, and the rheology of the spinning dope.^34^ The resulting highly viscous polymer solutions generate a core–sheath structure in the semicrystalline precursor fibers, depending on the spinneret geometry and the spinning speed.^35^ This structure is generated by stretching to produce a high degree of polymer chain orientation and crystallinity or complex defect structures when stretched, and the elastic limits are exceeded. Suitable methods for evaluating these effects are small- and wide-angle X-ray scattering (SAXS/WAXS),^36,37^ scanning electron microscopy (SEM), computed tomography (CT), or μCT.^38^

This work presents a new cross-scale approach to evaluating carbon fiber structure. This is demonstrated along the manufacturing chain from porous PAN precursor fibers (pPF) to porous carbon fibers (pCF), where the particular challenge lies both in maintaining the porous structure throughout all process steps of thermal conversion and in ensuring that the fiber is sufficiently strong for processing in the stabilization and carbonization steps. To achieve this objective, electron beam (EB) irradiation was introduced as an additional process step in the conventional carbon fiber production route—not to improve mechanical properties but to tailor the precursor structure prior to stabilization and carbonization.^39^ Through EB treatment, free radicals are generated and cross-linking is induced in polyacrylonitrile (PAN), leading to a modified thermal stabilization behavior. It has been reported that EB-treated PAN requires approximately 10–20 °C lower temperatures to achieve radical concentrations comparable to those of untreated fibers.^40^ A direct correlation between radical concentration and the degree of stabilization has been demonstrated in prior work.^41^ As a result, reduced stabilization temperatures and shorter processing durations become feasible, which in turn decrease the fiber’s exposure to heat and mechanical stress—key contributors to pore collapse. Furthermore, it has been shown that EB-induced cross-linking under a nitrogen atmosphere restricts molecular mobility above the glass transition temperature of PAN,^39,40^ potentially supporting the retention of pores, particularly in the fiber core. Based on this understanding, an electron beam treatment was used to cyclize and cross-link the PAN for prestabilization, whereby the cyclization index was determined on the basis of spectroscopic methods and was found to be 54% for the subsequently thermally porous e-beam-treated stabilized fiber (piSF). The produced fibers were characterized by classical textile-physical investigations along the process chain, as well as by means of analyses with regard to structural changes and radial distribution of carbon crystallites, pores, and cavities, which were carried out using X-ray techniques, including WAXS and nanoscale X-ray tomography (XRM), as well as density measurements, and imaging techniques such as SEM with correlative Raman spectroscopy and *in situ* atomic force microscopy, as well as transmission electron microscopy (TEM) in selected areas (Region of Interest, RoI). This approach also allowed for more precise statements to be made about the pore distribution and interconnectivity of these porous fibers. These findings provide a framework for a deeper understanding of the structure–property relationships along the carbon fiber manufacturing chain in order to develop tailored pCF for CF-based supercapacitors. In principle, these can be used for energy storage applications, since the pore systems are accessible to electrolytes.^31^

## 2 Methodology/Experimental

### 2.1 Fiber Manufacturing

#### 2.1.1 Precursor Fiber Spinning

Fiber spinning of the precursor fiber was performed by using a wet spinning pilot plant (Fourné Polymertechnik GmbH, Alfter-Impekoven, Germany), which includes a coagulation bath, three washing baths, and drying and winding units. To prepare the spinning dope, 16 wt % of PAN was consecutively added to DMSO and stirred continuously. The spinning dope was then processed at 70 °C through a 50 μm filter. A spinneret with 1008 holes, each 70 μm in diameter, was used for the spinning process. The fiber formation was performed at 50 °C in a coagulation bath containing DMSO and water with a water concentration of 27 wt %, followed by three washing baths with pure water and increasing temperatures of 60, 70, and 80 °C, respectively. The jet stretch used in the coagulation bath was 1.4, and the total stretch was 4.0. Drying was performed at 57 °C.

#### 2.1.2 Electron Beam

The electron beam (EB) treatment of the PAN-based pPF was performed under a nitrogen atmosphere using the ELV-2 electron accelerator (Budker Institute of Nuclear Physics, Novosibirsk, Russia) to achieve a cross-linked and an electron-induced cyclized fiber (further termed porous irradiated precursor fiber, piPF). The process parameters were set at a 1.5 MeV electron energy and 1 mA current. To ensure that the temperature increase during irradiation remained below the glass transition temperature of the pPF, the treatment was conducted in two stages. In each stage, the fiber received a dose of 100 kGy, with a 15 min cooling interval at room temperature between the two steps. This resulted in a total dose of 200 kGy, with the irradiation conditions (energy and current) maintained constant throughout the process.

#### 2.1.3 Carbon Fibers

For the carbon fiber manufacturing, the electron beam cross-linked fibers were stabilized using a furnace with four separate heating zones (HORN Glass Industries AG) at temperatures between 250 and 290 °C (porous irradiated stabilized fiber, piSF). The fibers were lightly stretched at ratios ranging from 0.3% to 0.5%, while a higher stretch of 1.6% was applied in the postheating zone. In total, a residence time of 13 min was chosen for the first thermal treatment. Afterward, they were carbonized in a nitrogen atmosphere at temperatures up to 800 °C (porous irradiated carbon fiber, piCF) in a low-temperature furnace (HORN Glass Industries AG) with four connected and one separate heating zone. While lasting 5 min in total in the heated area, with the same amount of time in each zone, the fiber was relaxed by the application of a negative tension of 6.45%. Next to this, an internal reference standard PAN-based carbon fiber was used for XRD and TEM measurements.

### 2.2 Initial Fiber Characterization of Structural Properties

#### 2.2.1 Textile Physical Characterization

The diameter of the filaments of the embedded fibers was measured by an Axio Imager.M1m microscope (ZEISS GmbH, Jena, Germany). The fibers were embedded in a thermosetting resin, the cross-sections of the polished specimens were imaged, and the diameter was measured with an integrated measuring tool. Tensile strength, Young’s modulus, and filament fineness (*n* = 20) were measured on a single fiber tester (FAVIMAT, Textechno Herbert Stein GmbH & Co. KG, Mönchengladbach, Germany) in accordance with DIN EN ISO 5079 (tensile strength, Young’s modulus) and DIN EN ISO 1973 (fineness).

#### 2.2.2 Density Measurements

Fiber density was determined using a flotation method in which two liquids with densities lower or higher than the expected fiber density are mixed until a fiber sample shows no upward or downward movement within a defined time window, indicating an equilibrium between the densities of the fluid mixture and the fiber. A defined volume of this fluid mixture is then weighed, and the fiber density is determined from the mass-to-volume ratio of the mixture.

#### 2.2.3 FTIR Measurements

FTIR spectra were recorded at room temperature by using a Nicolet iS-50 spectrometer. Each spectrum was averaged over 64 scans with a resolution of 4 cm^–1^, covering a wavelength range of 4000–650 cm^–1^. Baseline correction of the raw data was performed using the “msbackadj” function in MATLAB R2022a, and the cyclization index (CI) was calculated using the “findpeaks” function based on eq 1:

1 $$CI=\frac{0.29\times \mathrm{Abs}\left(1590\right)}{\mathrm{Abs}\left(2242\right)+0.29\times \mathrm{Abs}\left(1590\right)}$$

Here, Abs(1590) represents the absorbance of C=N groups, and Abs(2242) corresponds to the absorbance of C≡N groups. The constant of 0.29 reflects the absorption ratio between these functional groups.

#### 2.2.4 BET Measurements

The porosity of the carbon fibers was analyzed using the Brunauer–Emmett–Teller (BET) method on an Autosorb-1 (Quantachrome, USA) with nitrogen gas as the adsorbate at 77 K. Adsorption isotherms were recorded over a relative pressure range from *P*/*P*_0_ = 1 × 10^–7^ to 0.98, covering micropores (>0.7 nm) and mesopores.

### 2.3 Correlative and Microscopic Analysis

#### 2.3.1 Scanning Electron Microscopy (SEM)

A GeminiSEM 460 (Zeiss, Oberkochen) with a double condenser and equipped with the NanoVP-Mode was used for the investigations. Fibers were placed at carbon pads for the fiber bundles and, on the other hand, embedded in epoxy resin for cross-sectional images. Images were taken using Inlens and SE2 detectors at magnifications ranging from 100× to 150 000×. The SE2 detector was used for topographical information on the fibers. The Inlens detector was used for surface-sensitive information at higher resolution (starting from 1000× magnification, applicable to all samples) and for the visualization of the smallest ridges and pores on the surface (piSF, piCF). Additionally, voltage contrast imaging was performed with the Inlens detector to see RoI at the fibers with different local charging behaviors (applicable to the piCF). The mean diameter of single filaments was calculated using ZEN software (Zeiss, Oberkochen) from at least 9 single measurements. For the cross-sectional images, the RoI was imaged in NanoVP Mode with the VPBSD detector and an acceleration voltage of 15 kV. Only by this high acceleration voltage could smaller pores be visualized, as the embedding material was covering the pores with a thin film, which probably resulted from polishing.

#### 2.3.2 Zeiss Crossbeam FIB with SEM

The samples were fixed on a silicon wafer, which was also suitable for FIB-SEM analysis. FIB-SEM imaging was performed using a Zeiss Crossbeam 550 microscope equipped with a Gemini 2 column, with the SEM operated at 1.5 keV EHT, and backscattered electrons were recorded at an energy-selective backscattered electron (EsB) detector with a 700 V grid voltage. The large cross-section was created using the Ga+ FIB accelerated at a 30 kV and a 30 nA beam current. Polishing of the cross-section was performed using a 3 nA beam current. The user interface employed for the automated 3D tomography was the Carl Zeiss Atlas 3D Tomography application, which consists of a dual 16-bit scan generator assembly to simultaneously control both the FIB and SEM beams and dual signal acquisition inputs. The slices were milled with a FIB beam of 30 kV and 1.5 nA current for the 3D tomography. The 3D tomograph (slice and view) was acquired with a 10 nm voxel size: in SEM, the *X*–*Y* resolution was 10 nm, and the slice thickness was also 10 nm.

#### 2.3.3 Correlative Raman–AFM Imaging

The samples were prepared for the correlative Raman–SEM study in such a way that they could be imaged well with both instruments. Individual fibers were fixed to a silicon wafer (Figure 3), which is suitable for both Raman measurements and SEM examination.

**Figure 3:**
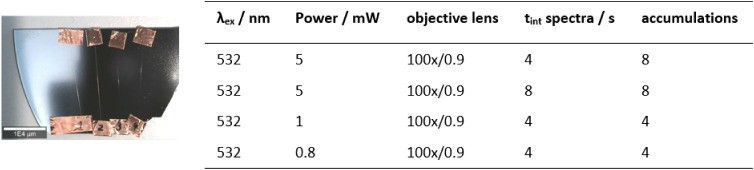
Sample settings and measurement conditions for correlative Raman spectroscopy.

An alpha300 RA Raman–AFM microscope (WITec GmbH, Ulm, Germany) with a 532 nm excitation laser was used. The laser power was adjusted by TruePower to 5, 1, and 0.8 mW, respectively. A 100×/0.9 objective (Zeiss, Germany) was used for the measurements, resulting in a diffraction-limited lateral resolution better than 300 nm. A back-illuminated CCD camera with 1600 pixels in combination with a UHTS 300 VIS spectrometer (WITec GmbH, Ulm/Germany) and a 600 g/mm (BLZ = 500 nm) grating was used to record the spectra. The correlative AFM images were obtained in Contact mode using contact mode cantilevers with a force constant of 0.2 N/m. To localize the same RoI within the alpha300 RA as in the SEM, the SEM images were subsequently correlated using TrueOrigin, a feature of WITec Control (WITec GmbH, Ulm/Germany).

#### 2.3.4 *In Situ* Atomic Force Microscopy (*In Situ* AFM)

The AFM measurements were carried out by AC-Modus (tapping or noncontact) to investigate the roughness (noise level: 25 pm). The deflection distance *z* of the tip was identified by using a reflective laser beam. The roughness is given by the root-mean-square (RMS, eq 2), which is the value of all ordinate values within the individual measurement range *l*. Also, the stiffness/hardness and scratch resistance of the single filaments were determined by the use of a cantilever (Adama Cone). The force constant used was 10 000 N/m.

2 $$RMS=\sqrt{\frac{1}{l}\int_{0}^{l}z^{2}\left(x\right)dx}$$

#### 2.3.5 Transmission Electron Microscopy (TEM)

The FEI Titan G2 Cube 60–300 instrument (FEI, Oregon/U.S.) was used for the examination. The investigated samples were the porous samples pPF, piPF, piSF, and piCF. In our work, the sample was cut perpendicular to the fiber axis using the FIB of the FEI-Helios SEM. Pore analysis was performed using ImageJ with a threshold set to detect voids larger than 16 nm.

#### 2.3.6 WAXS Crystallinity Tests

WAXS measurements were carried out using the laboratory SAXS/WAXS beamline Xeuss 3.0 (Xenocs, France) equipped with a D2+ MetalJet X-ray source (Ga Kα, 9.2 keV, wavelength [λ] = 1.341 Å) and an EIGER2 R 1 M detector. The X-ray source was operated at a voltage of 70 kV and a power of 250 W, or an anode current of 3.57 mA was generated. A sample detector distance of 70 mm was used. The examination time was 900 s and took place under a vacuum. Data reduction and analysis were performed using Xenocs XSACT integrated analysis software (XSACT 2.0). The data reduction among the equatorial direction was performed using a sector angle of 180 ± 10°, while the azimuthal profile covered 0–360° at the *q* range of 1.2 ± 0.1 Å^–1^ and 1.8 ± 0.2 Å^–1^ for pPF and piCF, respectively.

#### 2.3.7 Nanoscale X-ray Tomography (3D-Imaging, XRM Measurements)

Nanoscale X-ray tomography was performed on a ZEISS Xradia 810 Ultra Nano CT, which provides a spatial resolution of 50 nm (Table 1). The instrument is equipped with a 5.4 kV X-ray source. A phase ring is inserted in the beam path to achieve Zernike phase contrast, which leads to an edge enhancement and helps visualize small density variations in low absorbing materials. The data presented here were acquired in High Resolution Zernike Phase Contrast Mode with a 16.3 nm voxel resolution.

**Table 1:** Measurement Mode for X-ray Investigations

Mode	Mag	2D Res/nm	Voxel/nm	Field of View/μm
Large Field of View	200×	150	64	65 × 65
High Resolution	800×	50	16	16 × 16

A single fiber was glued at the tip of a steel pin using UV-cured adhesive for nondestructive investigation. A series of 301 X-ray projection images with a 16 μm field of view were collected as the fiber was rotated over a 180° rotation span. The acquisition time for each projection was 400 s. Volume data were obtained by processing the projection data with ZEISS Scout & Scan reconstruction software.

## 3 Results and Discussion

### 3.1 Initial Fiber Properties and Surface Morphology

The manufacturing process of carbon fibers involves at least three essential steps: precursor production, oxidative stabilization, and carbonization in an inert gas atmosphere.^42^ In most cases, surface functionalization is also carried out. In the case of porous precursor fibers (pPF), there is also the step of electron beam treatment, which was applied after the precursor spinning to preserve the developed pore structure along the thermal conversion processing steps, as Gohs et al. have shown.^43,44^ Here, with respect to the surface morphology, as measured by SEM, no major changes were observed in all of the processing steps. The initial striation of the stretching of the PF is shown in Figure 4A,B and was kept over all other processing steps: the cross-linked and cyclized piPF (Figure 4C,D), piSF (Figure 4E,F), and piCF (Figure 4G,H). However, each type of fiber has its own characteristic appearance, according to visible pores or morphology. The piPF and piCF have pores with an elongated structure, smaller pores, and irregularities (Figure 4F). In particular, piCF has a rougher morphology.

**Figure 4:**
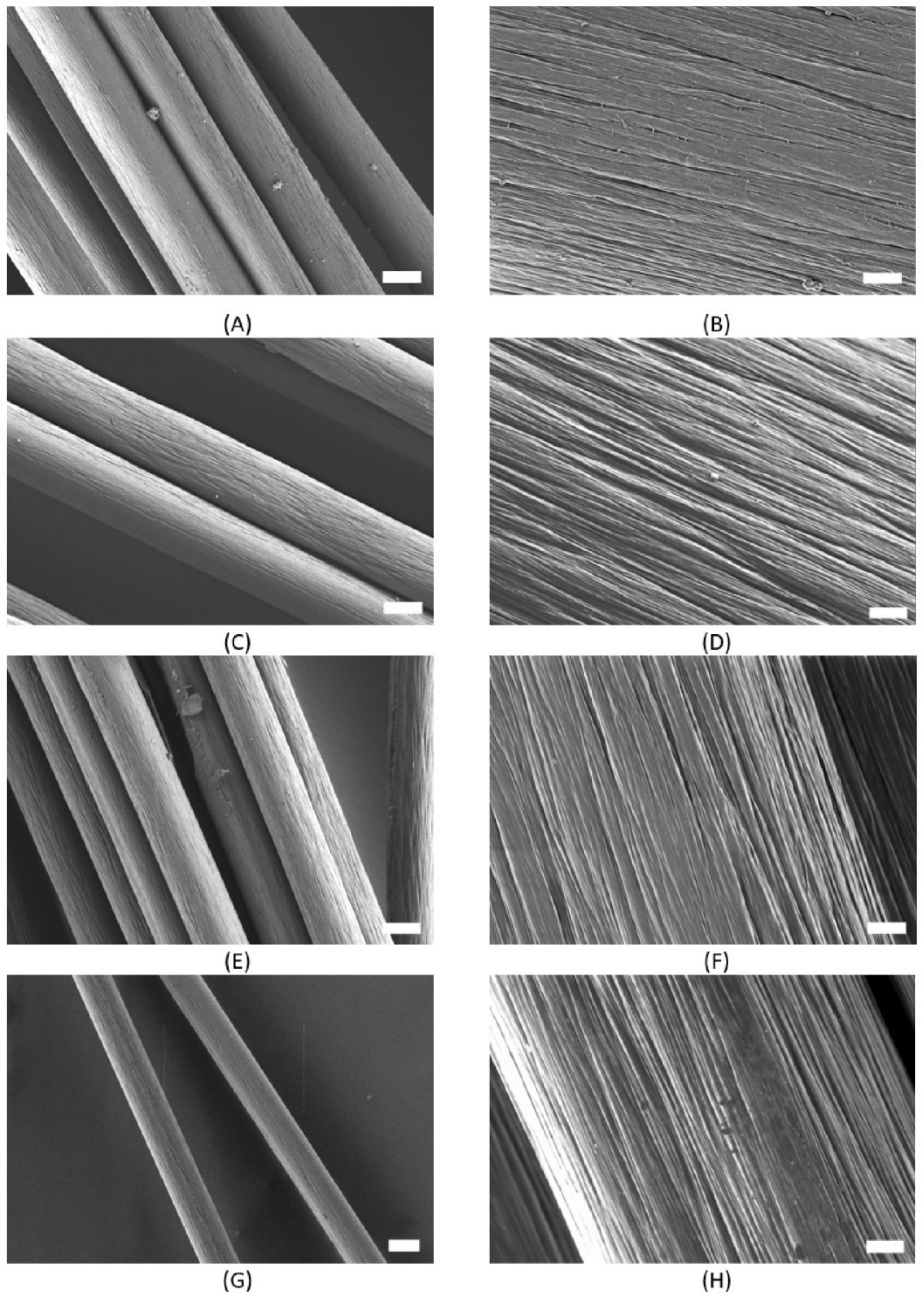
SEM images of (A) scale 10 μm (magnification 1000×) and (B) scale 1 μm (magnification 10 000×) precursor fiber, (C) scale 10 μm (magnification 1000×) and (D) scale 1 μm (magnification 10 000×) e-beam cross-linked fiber, (E) scale 10 μm (magnification 1000×) and (F) scale 1 μm (magnification 10 000×) stabilized fiber, (G) scale 10 μm (magnification 800×) and (H) scale 1 μm (magnification 10 000×) (with in-lens detector) carbonized fiber; the overview measurements were done with an EHT of 0.8 kV at high vacuum.

However, when looking at the cross-sectional images (Figure 5), a homogeneous structure is visible for the pPF as well as the piPF (Figure 5A,B). This supports the assumption that electron beam treatment^43,44^ results in a uniform change in properties because it goes right through the fiber. Despite this, the highest measured surface area was found for the pPF with 39.95 m^2^/g (Table 2), which is less than 70 m^2^/g as we could demonstrate for previously prepared porous PAN-based precursor fibers.^45^ After the e-beam treatment, the measured surface area decreased to 36 m^2^/g. However, the rough and open-pore surface morphologies shown in Figure 4 suggest that the surface structures are accessible for both fiber types. It was then reduced to 0.99 m^2^/g after stabilization and carbonization. Here, also the surface morphology appears smoothened (Figure 4E–H).

**Table 2:** Diameter of the Investigated Fibers by SEM* and LM** as Well as Textile Physical Properties and BET Measurements

Fiber	Diameter/μm*	Diameter/μm**	Density/g/cm^3^	Tensile strength/MPa	Young’s Modulus/GPa	BET surface area/m^2^/g
pPF	18.38 ± 1.75	13.45 ± 0.5	1.159 ± 0.006	318 ± 17	9 ± 0.39	39.95
piPF	17.92 ± 0.99	14.12 ± 0.65	1.156 ± 0.025	244 ± 9	8 ± 0.23	36
piSF	13.74 ± 1.03	13.8 ± 0.36	1.333 ± 0.005	166 ± 6	7 ± 0.23	0.63
piCF	10.70 ± 0.93	10.69 ± 0.38	1.537 ± 0.04	649 ± 59	45 ± 1.8	0.99

**Figure 5:**
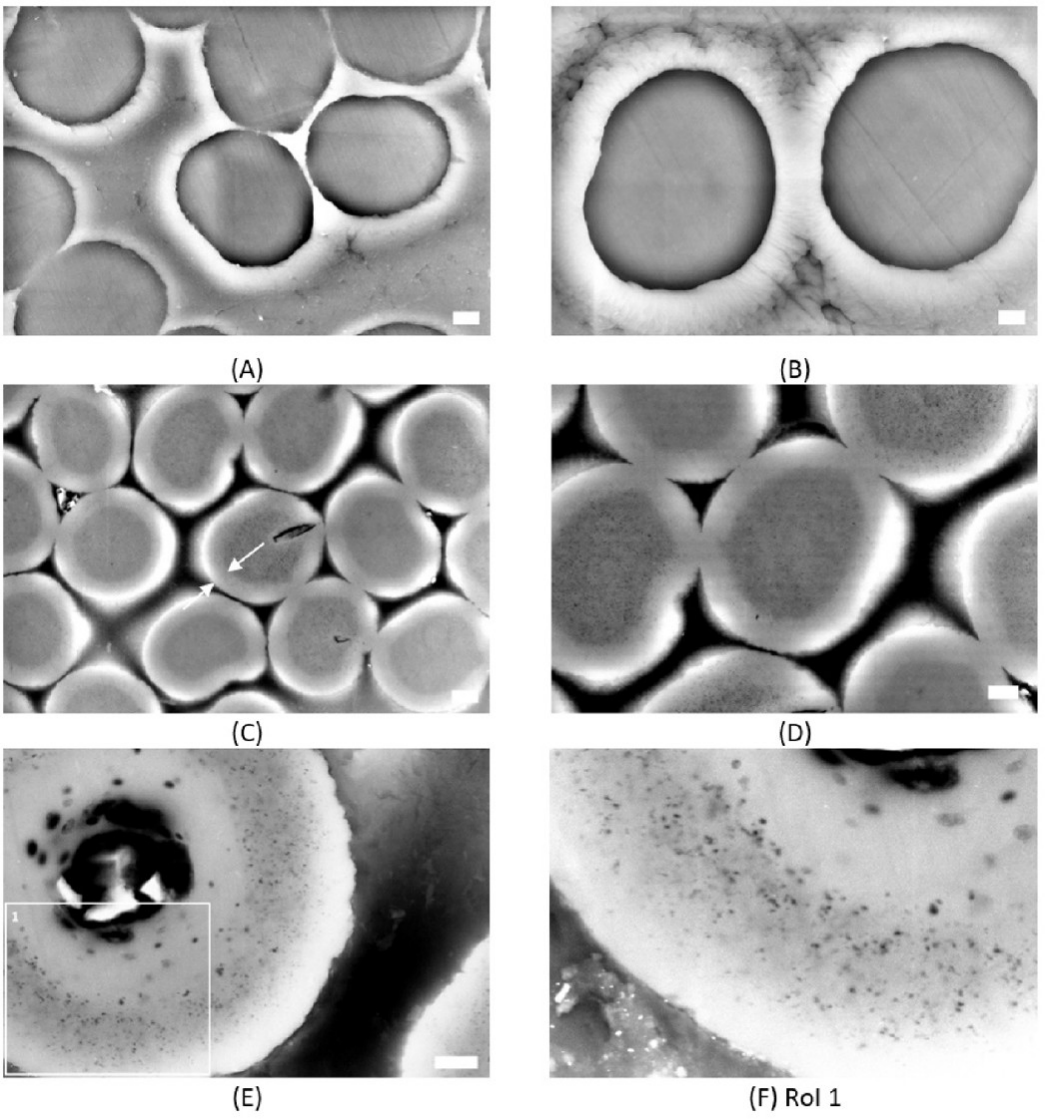
SEM images were taken with 15.00 eV at the cross-section prepared using embedded fibers in epoxy resin with subsequent grinding: (A) precursor fiber with a scale of 3 μm, (B) e-beam cross-linked fiber with a scale of 2 μm, (C) stabilized fiber with a scale of 3 μm, whereas the mean of the standout sheath structure (white arrows) is 2.14 ± 0.19 μm (*n* = 16), and (D) stabilized fiber with a scale of 3 μm as well as (E) carbonized fiber with RoI 1 with a scale of 1 μm and RoI 1 of carbonized fiber with a scale of 300 nm (F).

Looking at the cross-section of the fibers (Figure 5), for the pPF, the filaments exhibit a round shape next to some kidney formed (Figure 5A). Across the filament, no particular morphology can be seen, as mentioned before also for piPF (Figure 5B). A core–sheath structure is visible for the piSF as well as for the piCF (Figure 5C–F). But the structure of the two forms differs significantly. The piSF shows a uniform distribution of fine pores in both the outer ring and the core. Basically, this core–sheath structure can be attributed to the stabilization process. It is known that there is better accessibility for oxygen in the outer area of the filaments, and consequently, a very well-stabilized and oxidized region exists (here, approximately 2.14 ± 0.19 μm (*n* = 16), Figure 5C). However, a so-called skin forms here, so that the oxygen or oxygen species can only diffuse into the core with difficulty, and the reactions take place much more slowly here, so that only a mainly stabilized region is created in the core due to diffusion.^42,46^ During carbonization, the cross-section changed dramatically. The core–sheath structure is still present, but there is a small ring of small pores and larger pores in the center (Figure 5G) with sizes up to 2.81 ± 0.32 μm (*n* = 4) (Figure 6). As can be seen from the different cross-section images obtained by SEM—once in a polished cross-section with filaments embedded in epoxy (Figure 5) and once in a FIB preparation, both methods have advantages. In the traditional polished cross-section, the resulting core–sheath structure can be seen better, whereas the contour sharpness of the somewhat larger pore structure can be seen better in the FIB (Figure 6). But in general, a lower acceleration voltage produces a better image quality with both.

**Figure 6:**
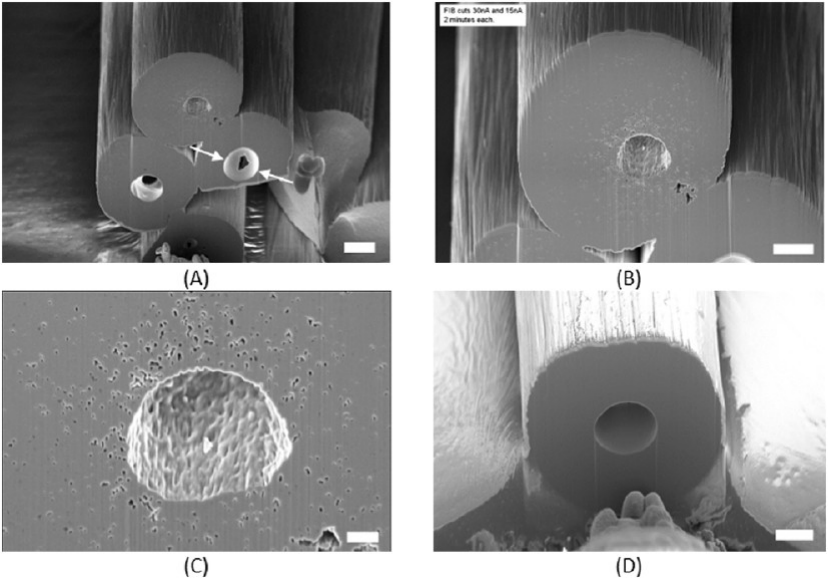
FIB piCF InLens image: 54° table tilt, cross-section (A) with a scale bar of 3 μm, prepared with 30 nA and 15 nA; (B) with a scale bar of 2 μm, InLens image after 3 nA FIB polishing, where large pores have a diameter of 2.81 ± 0.32 μm (*n* = 4); (C) with a scale bar of 500 nm; and target site (FIB cross-section) for 3D tomography with a scale bar of 2 μm (D).

The initial fiber properties are shown in Table 2. The textile physical fiber properties of all porous fibers are comparatively low: starting with 318 ± 17 MPa for the pPF, they decrease to ∼77% for piPF and piSF to ∼52%, followed by an increase for the final piCF with 649 ± 59 MPa. The Young’s modulus for the piCF of 45 GPa is also well below generally known material data for PAN-based CF, which are around 200–300 GPa.^1^ The aim of this work was not to obtain a CF with high mechanical strength but to demonstrate that an e-beam treatment can be used to preserve a pore structure created in the precursor throughout the further process steps and that the structure can be intensively described using suitable high-resolution analytical methods in order to achieve a better understanding between morphological and intrinsic properties.

The density of the obtained porous fibers shows an increase from the pPF at 1.16 g/cm^3^ to the piCF at 1.54 g/cm^3^ (Table 2). The density of the piCF is still below the density reported in the literature for PAN-based CF ranges from 1.76–1.91 g/m^3^,^1,2^ so it can be assumed that this is caused by an intrinsic porous structure, which could be demonstrated by FIB (Figure 6), cross-sectional SEM (Figure 5), and XRM measurements (Figure 13). Despite the pores and voids, the structure of piCF is more compact, whereas the density increases due to the carbonization process with its loss of H-atoms and building of graphitic structure.

With regard to the surface morphology, further investigations were carried out using *in situ* AFM (Figure 7). This allows continuative impressions of the surface topography to be combined (Figures 4 and 7) and quantified by means of accompanying roughness and indenter measurements (scratch tests on individual filaments). The topographical scans for the piCF showed the highest visible roughness (Figure 7H) but were only determined with an RMS of 18 nm, as was the piSF with an RMS of 18 nm. For the pPF, the RMS was 19 nm, and for the piPF, the RMS was 16 nm. The pPF and the piPF (Figure 7B,D) show as pure polymer-based fiber (coPAN) strong indentations, the piSF (Figure 7F) shows lighter scratches, and the piCF (Figure 7H) shows no or only minimal changes due to the consolidated structure by thermal stabilization and carbonization. Next to this, the piCF shows a rougher surface, and together with the piSF, they showed greater scratch resistance as mentioned before.

**Figure 7:**
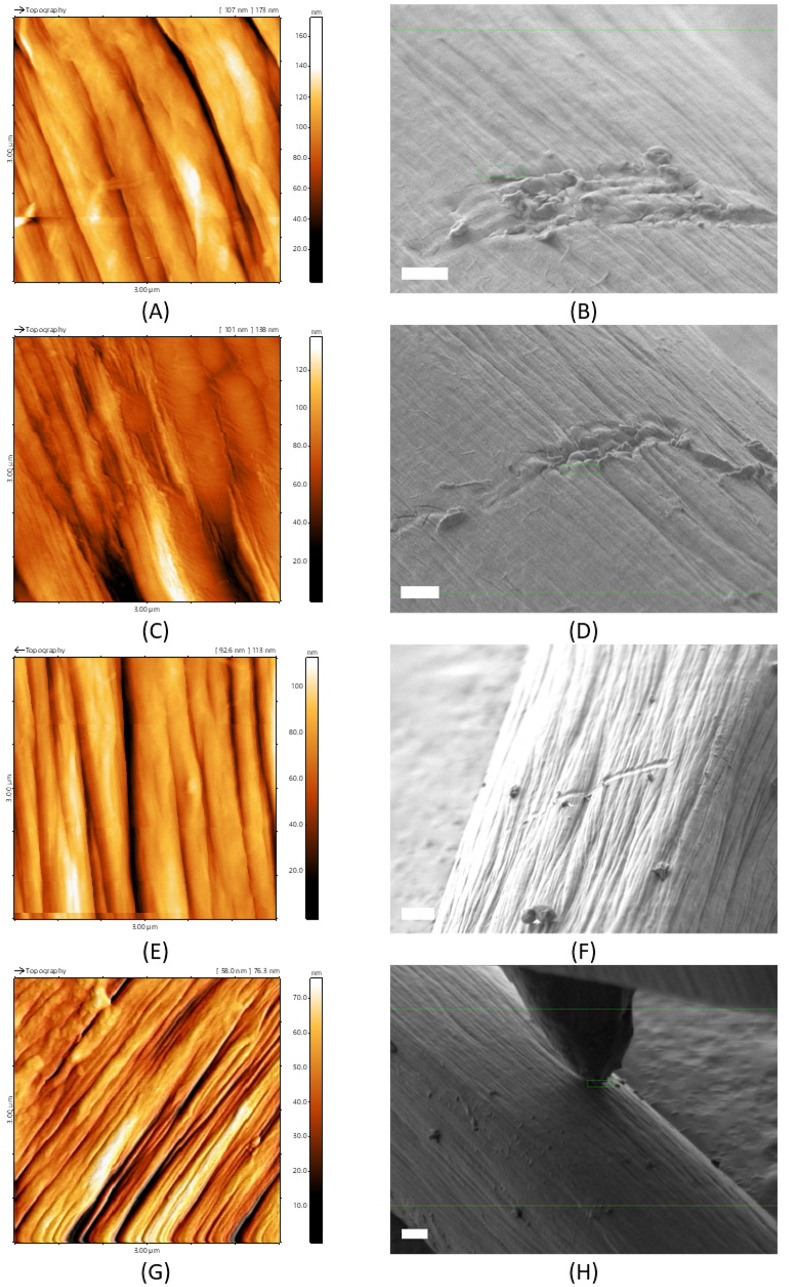
AFM topography images of the RoI of the investigated fibers: pPF (A), piPF (C), piSF (E), and piCF (G), and *in situ* AFM scratch test using an AFM tip “Adama Cone” (Adama Innovations Ltd., Ireland) with a force constant of 10 000 N/m for observing using SEM Zeiss Gemini460: pPF (B), piPF (D), piSF (F), and piCF (H) with a scale bar of 1 μm.

### 3.2 Spectroscopic Measurements and Correlative Raman Measurements

As described in the literature, an e-beam treatment results in both cross-linking and cyclization of PAN-based materials.^43,44^ In this respect, FTIR measurements can be used to determine a cyclization index (CI) both after e-beam treatment and after oxidative stabilization, thus providing proof of the success of the e-beam treatment. This is done by taking into account the specific bands of the nitrile groups (C≡N) and the imine groups (C=N) (Figure 8A). But, the spectra of the pPF and the piPF (200 kGy) initially show no significant differences (only a slight increase of the CI), indicating that e-beam irradiation does not significantly alter the primary chemical groups in the PAN structure. In contrast, the piSF exhibits a prominent peak at 1590 cm^–1^, corresponding to the formation of C=N. This suggests cyclization reactions, where C≡N bonds are converted into conjugated cyclic structures containing C=N bonds during thermal stabilization. The CI (Figure 8B) further emphasizes this trend. While e-beam treatment does not affect the intensity of C≡N or promote cyclization, thermal treatment results in a substantial increase in the CI. This increase, to 54%, confirms the development of cyclic structures in the piSF, a critical step in the stabilization of PAN-based materials.

**Figure 8:**
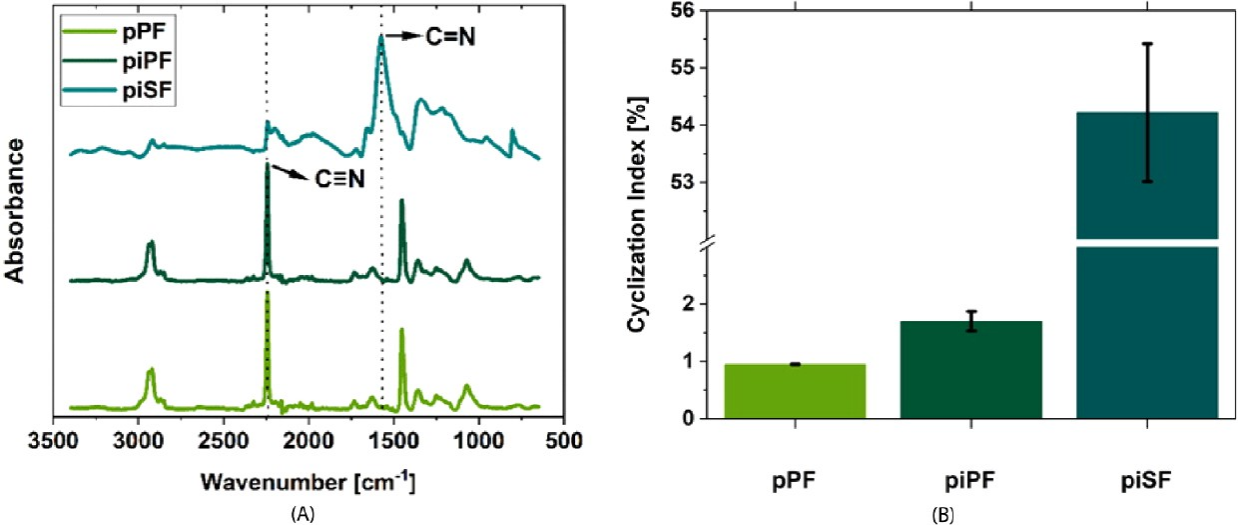
FTIR spectra (A) and relative cyclization index (CI) (B) of pPF, piPF, and pSF.

Correlative measurements using Raman, AFM, and REM were also carried out for the structural investigations. For the correlative measurements, all samples were investigated by using SEM and subsequently analyzed by using Raman spectroscopy. The correlative process is exemplarily shown in Figure 9. Correlative Raman images were performed as a stand-alone combination but can also be carried out *in situ*. For the pPF and piPF, the Raman depth measurement shows the profile of the fiber in the range of the penetration depth of the laser.

**Figure 9:**
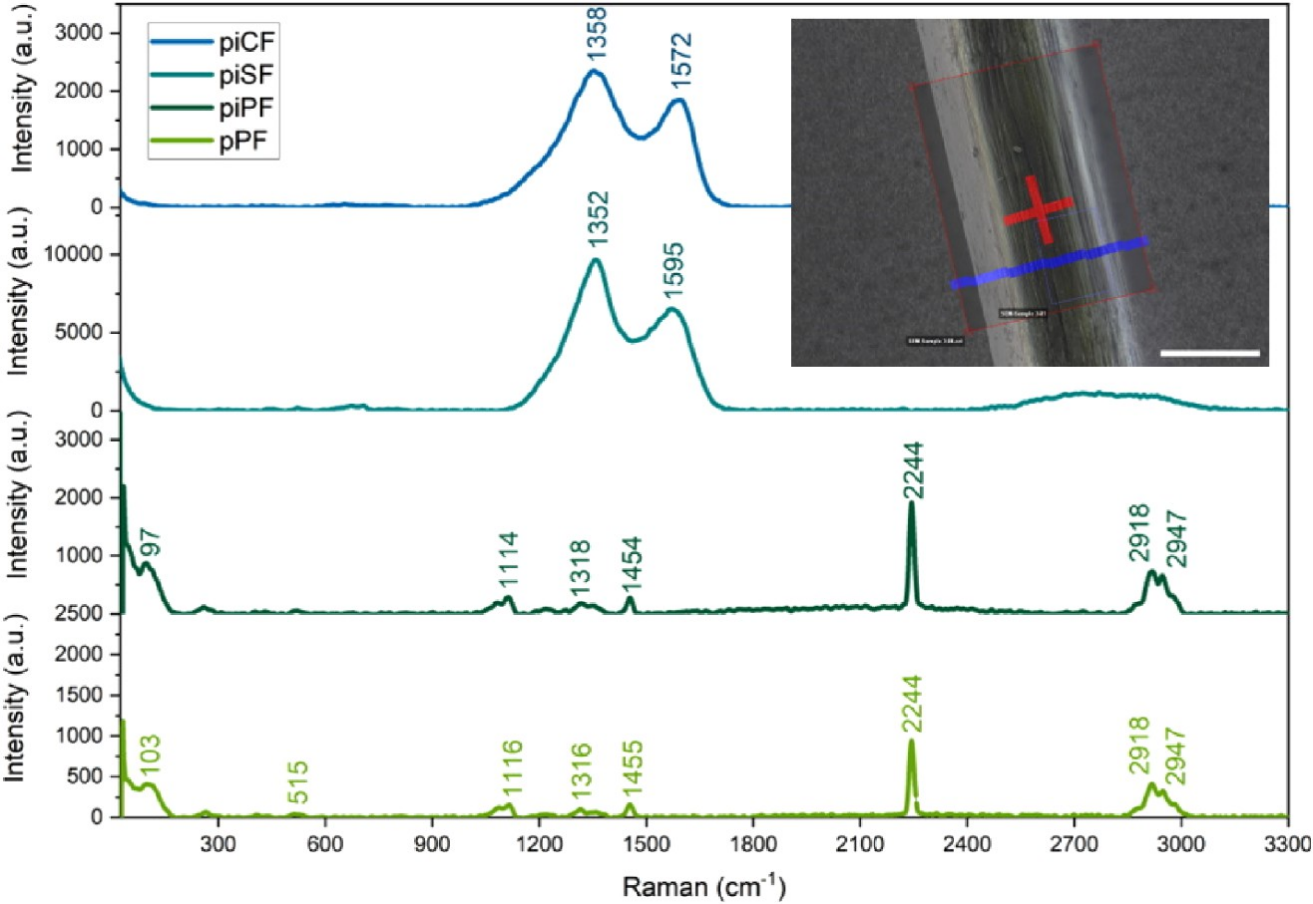
Presentation of the Raman site using SEM recovery using the correlative workflow demonstrated for the piSF (SEM image) with a scale bar of 10 μm and Raman spectra of investigated filaments; position of single spectrum measurements (red) and depth scan area (blue). Similar methods were applied for all fiber samples.

In particular, Raman measurements enable a more precise description of the carbon structures formed. These Raman measurements can distinguish between the PAN structure and the cross-linked/electron-induced cyclized PAN structure and the sp^2^-dominated carbon, which show the typical D peaks (1350–1360 cm^–1^)—associated with a disordered diamond-like carbon structure—and G peaks (1580–1590 cm^–1^)—ordered carbon clusters—,^47–49^ Furthermore, the ratios of the areas (*I*_D_ and *I*_G_) under the bands (*I*_D_/*I*_G_) provide information about the amorphities of the examined filaments. The larger the ratios, the more defects are contained in the structure and ordered clusters.^50,51^ This could be shown for the piSF as well as for the piCF fiber with *I*_D_/*I*_G_ ratios of 1.7 and ∼3.0 (Table 3). The in-plane graphitic crystallite size *L*_a_ value was inversely proportional to the *I*_D_/*I*_G_ value. Also, the mole fraction of graphite *X*_G_ value of the piSF is higher at 36.2% compared to the piCF at 24.5%. Next to this, the piCF also exhibits a distinct disorder-structure peak (A peak), which can be seen in detail in Figure 10B.

**Table 3:** Detailed Peak Parameters of the piSF and piCF Obtained from Fitted Raman Curves with Full Width at Half Maximum (fwhm) and *L*_a_ = 4.4/(*I*_D_/*I*_G_) and *X*_G_ = (*I*_G_ × 100/(*I*_G_ + *I*_D_)) according to Wang et al.^55^

	*I*_D_/*I*_G_	*L*_a_/nm	*X*_G_/%	D peak fwhm/cm^–1^	G peak fwhm/cm^–1^	A peak fwhm/cm^–1^
piSF	1.763	2.50	36.187	180.69	138.67	-
piCF	3.083	1.43	24.493	240.98	98.27	64.96

**Figure 10:**
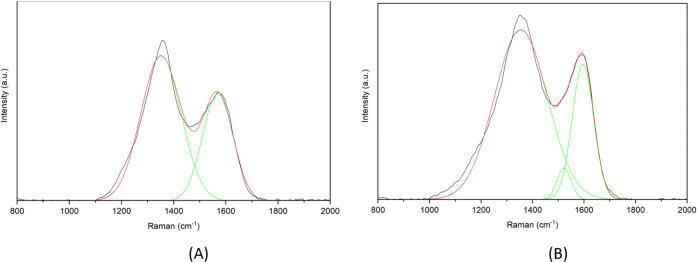
Raman spectrum of the porous stabilized (A) and carbonized fiber (B) (fitting curves green).

The pPF and piPF are very similar in terms of the structures shown in the Raman spectrum (Figure 9). Both show fluorescence but can still be measured well with 532 nm excitation and exhibit the typical bands of PAN. These are mainly the C≡N stretching vibrations at 2244 cm^−1^ ^52^ and the CH_2_ bending (scissoring) at ∼1455 cm^–1^, as well as a typical PAN shoulder at 1116 cm^–1^ of the raw PAN fingerprint region. This is accompanied by a band for aromatic stretching vibrations at 2918 cm^–1^ and aromatic vibrations at ∼1316 cm^–1^, the appearance of which may be due to the fact that the original material is not a homo-PAN.^53^

The piSF and piCF show a clear change (Figure 10): the two spectra show small differences in the shape and ratio of the peaks. The piSF shows fluorescence. For piSF, as in the single spectrum, only carbon can be observed in the spectrum of this sample. However, there are slight differences in the spectra between the surface and the underlying layer. The D peak (1358 cm^–1^) is slightly narrower compared to the piCF with the peak at 1352 cm^–1^ for piSF, which led to the consumption of a higher degree of crystallinity^54^ for the stabilized sample. The G peak was obtained at 1568 cm^–1^ for the piSF and 1595 cm^–1^ for the piCF.

There are also differences in the morphology that are recognizable. No significant spectral differences are visible throughout the measurement range (Figure 11A,B). The cross-section is no longer round but has indentations (Figure 11C,D). Compared to the piSF, only one spectrum can be observed in the piCF. A special characteristic of piSF is that the individual spectrum differs from the spectrum analysis of the deep scan (Raman, Figure 11C blue). This can probably be attributed to the core–sheath structure already observed in the SEM cross-section (Figure 5C,D). Compared to the other measurements, the spectra differ in composition. Here, the D-band of the deeper layer is thinner, compared to the surface-close layer (Figure 11F). The sum spectrum was used to display the Raman spectra in Figures 9 and 10A. As with the single spectrum, only carbon can be observed in the spectrum of this sample. However, slight differences can be observed in the spectrum between the surface and the layer directly below it. The D peak (approximately 1350 cm^–1^) is slightly narrower here.

**Figure 11:**
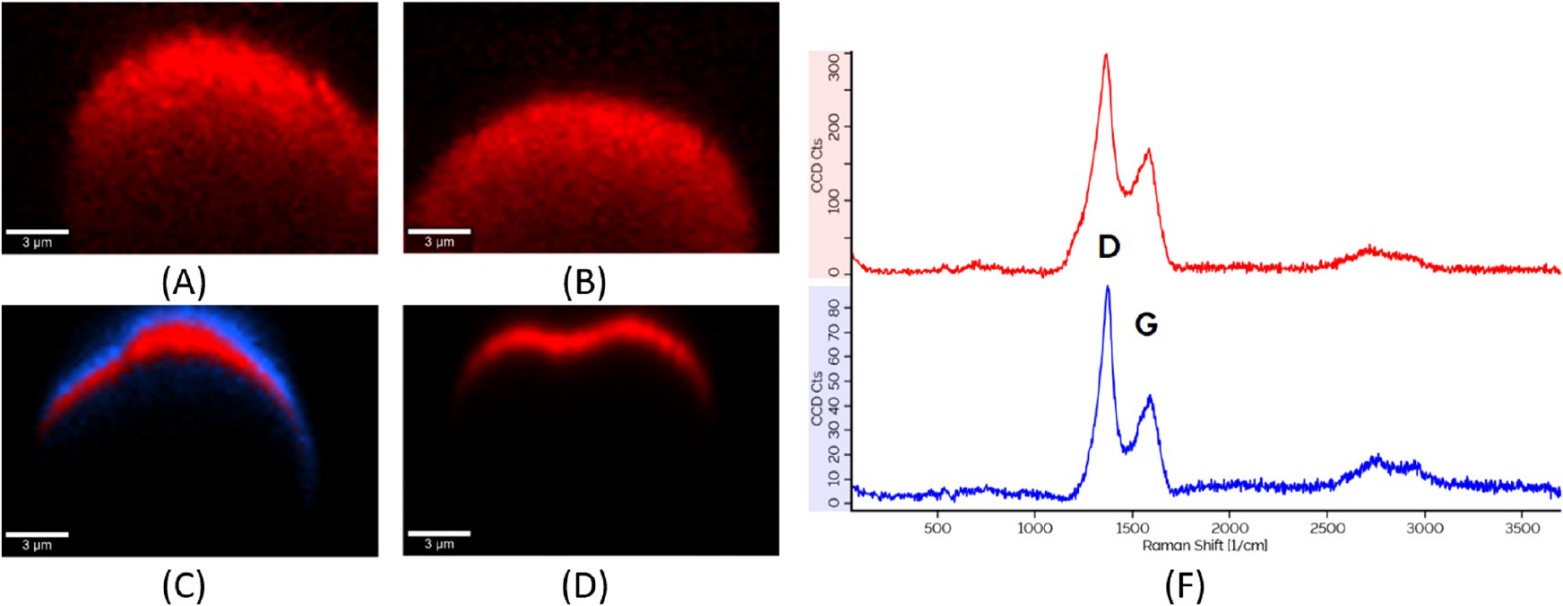
2D-Raman depth measurement as a color-coded Raman image: pPF (A), piPF (B), piSF (C), and piCF (D), with areas of single measured spectra (red) and depth scan (blue) (F). Only for the piSF, a significant difference between single measurements and depth scan was visible. The evaluation was carried out using a true component analysis.

In addition to the Raman spectra, correlative mapping was performed for SEM and AFM measurements of the same RoI. This was done for both piSF (Figure 12A–C) and piCF (Figure 12D–F). For the piSF, small pores are visible in the centers of the fiber cross-sections. The topography AFM image shows differences in the height level (Figure 12B), with the outer part of the fiber being stiffer compared to that of the inner core. Due to the mentioned difference in hardness, the material is removed differently during grinding at the preparing of the polished sample with the embedded fibers. A difference in the Raman signal between the sheath and the core of the fiber was observed and is displayed in Figure 12C. For the piCF, small nanopores are visible in the outer region. The fiber core appears partially hollow, although the topography image shows no differences in the height level of the sheath.

**Figure 12:**
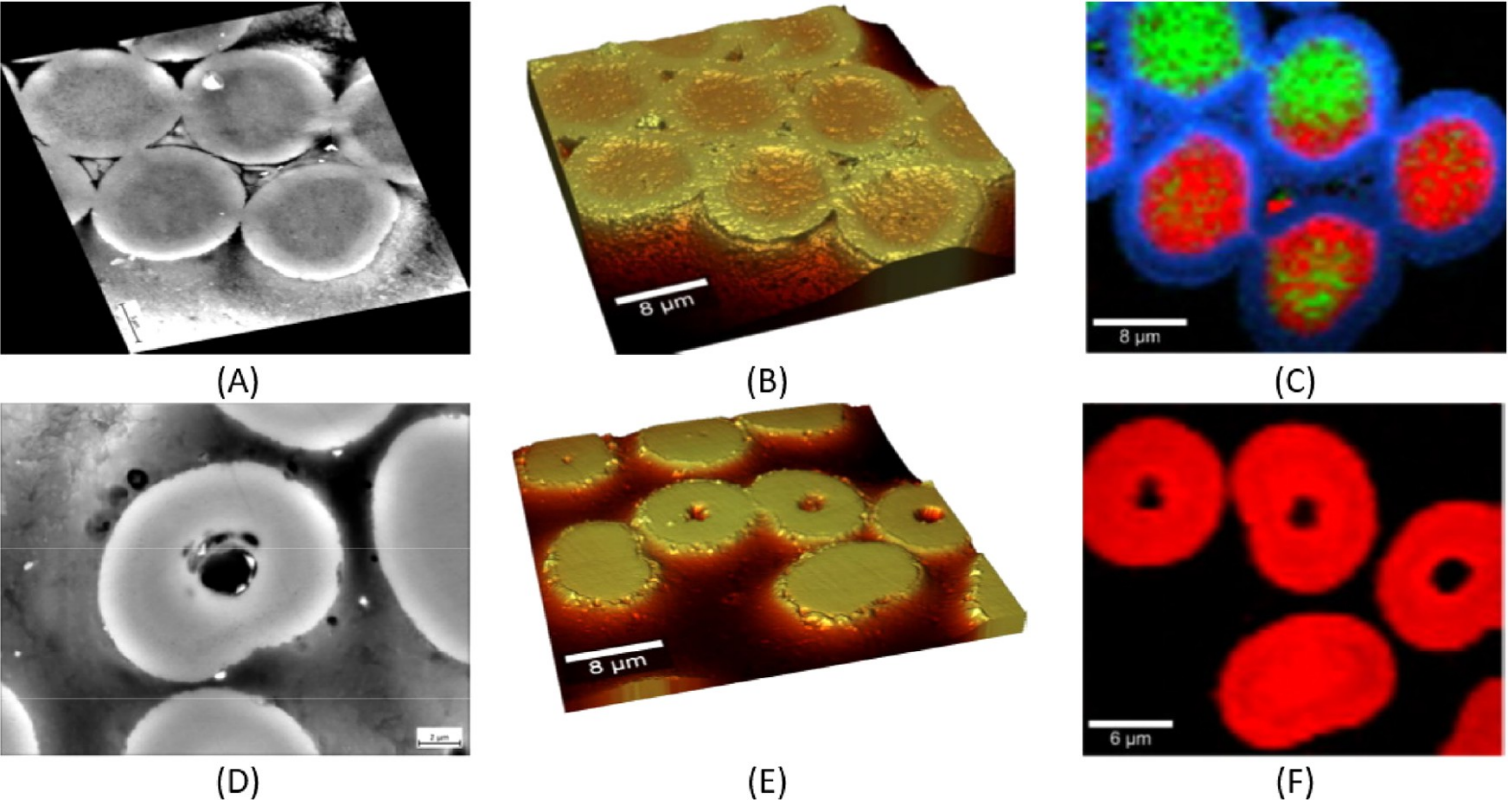
Correlated RoI for SEM (A), AFM (B), and Raman mapping (C) image of the piSF and SEM (D), AFM (E), and Raman mapping (F) image of the piCF; the colors of Raman mapping (C) and (F) are referred to the different phases and spectral compositions. For red, the sample is homogeneous, and no spectral difference could be seen.

### 3.3 3D X-ray Microscopy

As already demonstrated in the preceding imaging and structural analysis methods, the structure of the final piCF obtained proves to be quite complex and heterogeneous. For example, to determine the size of the large pores in the core area of the fiber, 3D X-ray microscopy (XRM) is very informative. Figure 13A shows this structure, and it can be seen that it is not a hollow fiber, as might be assumed at first inspection of Figure 5E (SEM cross-section), Figure 6 (FIB), and Figure 12D (SEM cross-section) but that there are several pores of different sizes distributed along the fiber’s central axis and core, respectively. Especially with respect to the promoted low tensile strength as a macroscopic property in Table 2, the results according to the different sizes and distribution of pores gain a realistic appreciation according to the found microstructures, which can be further complemented with the TEM measurements. This demonstrates the importance of the combination and correlation of high-resolution analysis methods to ensure a comprehensive understanding. In order to obtain a better image, a KI-assisted reconstruction process (Deep Recon dataset) was used to improve the contrast (Figure 13) to reduce the noise and make it easier to segment the data and generate the volume plot (Figure 13C).

**Figure 13:**
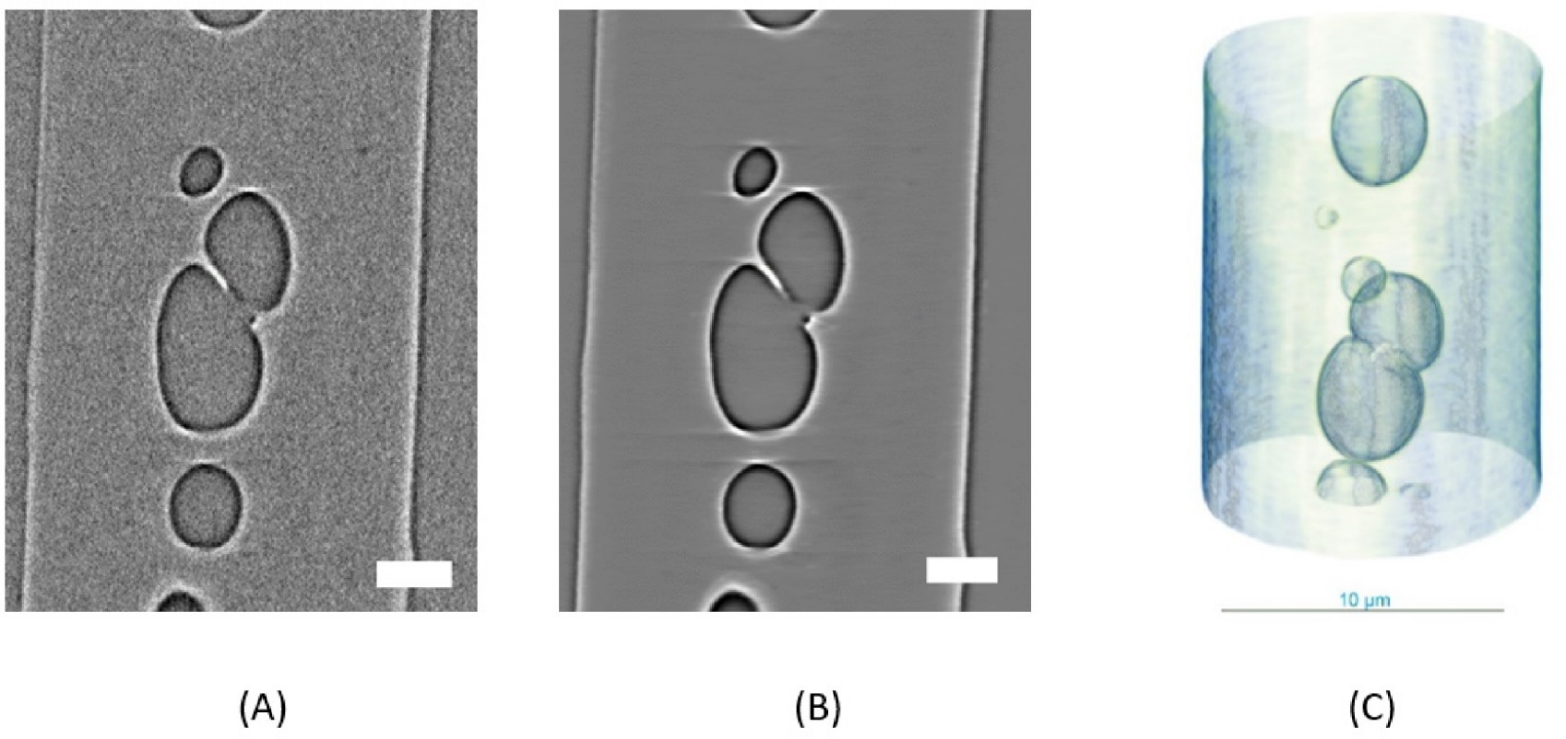
Virtual cut of a piCF phase contrast as a standard image (A) and an image corrected using noise canceling (Deep Recon Pro) (B) with a scale bar of 2 μm; resolution of 16 nm for each voxel and final nondestructive 3D mapping of the fiber structure (C).

### 3.4 Transmission Electron Microscopy

Due to the reason that the defects, voids, and pores occurring in fibers and CF are quite small, TEM investigations were also carried out. It was found that all the fibers examined exhibit nanoscale porosity, as already indicated by the cross-section SEM (Figure 5) and FIB (Figure 6) investigations. Each sample provides a distinct distribution of pores in a differently shaped structure (Figure 14A–E). Figure 14a–d shows a stacked pore distribution because it was measured using a TEM. Based on the stacked pores, it is judged that there is a possibility that the propagation was made in one pore.

**Figure 14:**
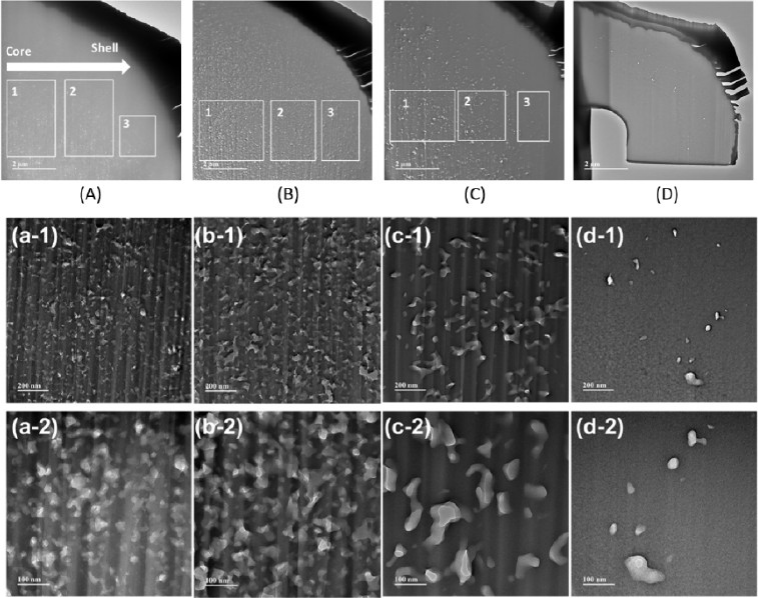
TEM cross-section of (A) pPF, (B) piPF, and (C) piSF with designated RoI; (D) piCF and TEM measurements of magnified cross-sections of (a-1/a-2) pPF, (b-1/b-2) piPF, and (c-1/c-2) piSF with designated RoI and (d-1/d-2) piCF. Here, the lamella cut direction can be seen in the images, especially forms a–c next to the depth effect, which leads to an optical overlay of the pores.

As seen in Table 4, pPF, piPF, and piSF have different pore distributions within the fibers from piCF, which also could be stated with the previous measurements by Figure 5E (SEM cross-section), Figure 6 (FIB), Figure 12D (SEM cross-section), and Figure 13 (XRM). The pPF and piPF have more pores in the core than in the shell of the fiber, wherefore it can be assumed that the achieved main pore structure could not brought through the stabilization processing step. The piCF has porosity in the midcore and in the sheath of the fiber.

**Table 4:** Summary of the Pore Distribution of the Investigated Fibers pPF, piPF, piSF, and piCF Initial Assessment by the Investigator

Sample	Core	Midcore	Shell
pPF	Porous	Porous	
piPF	Porous	Porous	Porous
piSF	Porous	Porous	
piCF		Porous	

According to the RoI in Figure 14, the pPF, the piPF, and the piSF have pores in both the core and the shell, as shown in Figure 15. There is a tendency that pore size and the number of pores were decreased from RoI 1 (core) to 3 (sheath) in the case of all the fiber samples.

**Figure 15:**
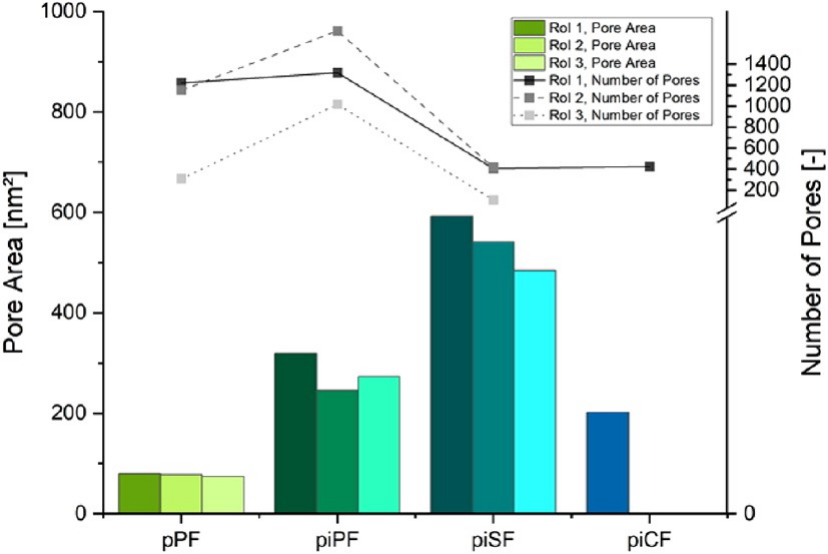
Measured pore area (bar chart) and pore size (lines) at the different RoI of pPF, piPF, piSF, and piCF; the color intensity decreases with the RoI position from the inner part of the fiber to the outer RoI (brighter).

The piSF has the largest pores out of all of the fiber samples, yet it also has the fewest pores in total. This led to consumption as smaller pores merged during thermal stabilization.^56^ The piCF has pores in the midcore and the sheath as shown in Figure 15 (bottom). For CF in general, smaller and larger numbers of the voids were created in the sheath of the fiber.

### 3.5 WAXS Crystallinity Tests

In addition, WAXS crystallinity measurements were carried out to provide comprehensive structural clarification. These, together with the previously presented Raman results, can provide information about the C-structures formed. Figure 16 shows a symmetrical arrangement of the reflexes with a 2-fold symmetry, which leads to the assumption of an alignment in a preferred direction, presumably in the fiber axis caused by fiber manufacturing. This is particularly evident in samples (A) and (B). With increasing temperature treatment, this evens out: stabilization of piSF (C) and carbonization in the piCF (D) and reference fiber (E). In the case of the piSF (C), an accumulation is still visible in the overall area and an orientation is present but without a preferred direction. In the case of the piCF (D) and CF (F), the detected signal is more diffuse, so it can be assumed that the crystalline areas are few and have different spacings or that there are also many interfaces, which indicates amorphous phases (disturbances, bremsstrahlung). The analysis of the Herman factor of the (002)^57,58^ peak revealed that the pPF had a value of 0.20 and maintained at 0.19 (piPF) before it decreased to 0.06 for piSF. Afterward, the Herman factor dramatically increased to 0.49 for the internal reference PAN-based CF. This result indicated the realignment of carbon chains along the fiber direction.

**Figure 16:**
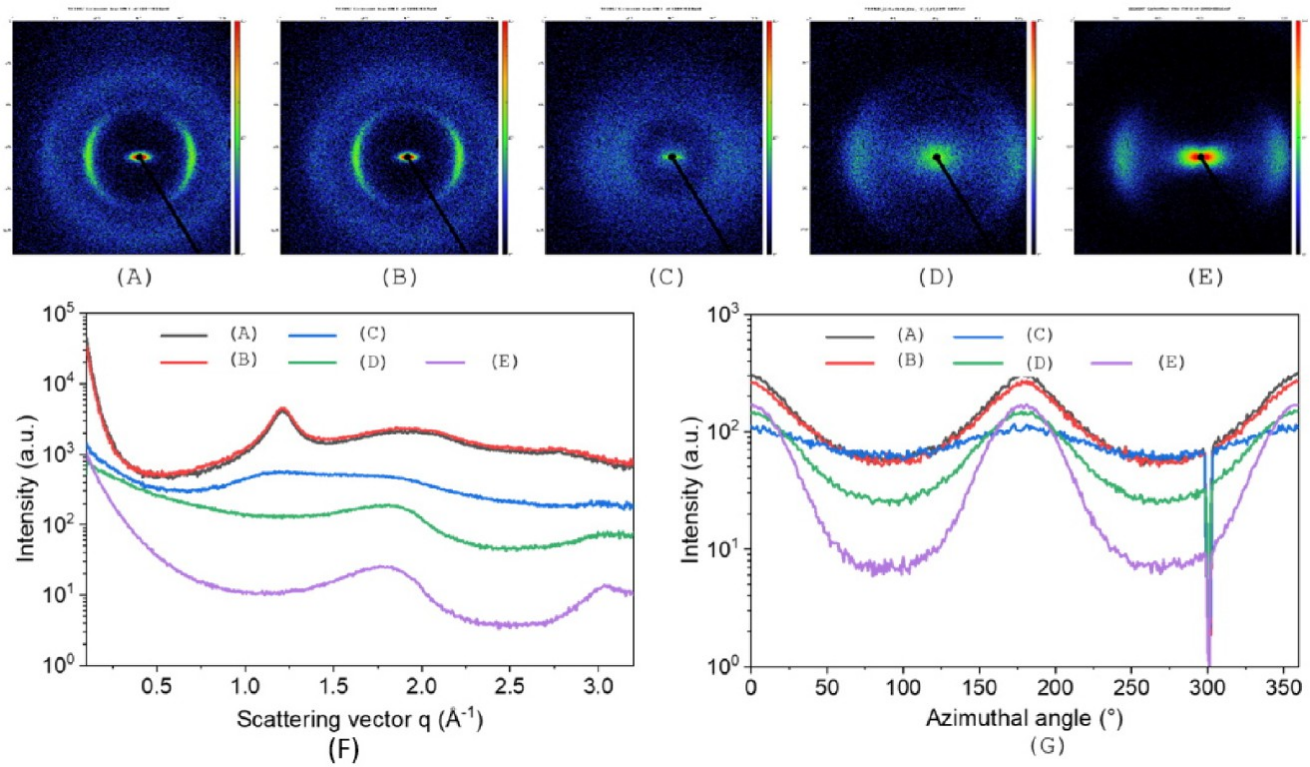
2D WAXS patterns of (A) pPF, (B) piPF, (C) piSF, (D) piCF, and (E) internal reference PAN-based CF. The intensity profile as a function of (F) scattering vector *q* and (G) azimuthal angle at the (002) peak position.

## 4 Conclusion

A porous PAN-based carbon fiber was prepared from a porous precursor fiber, which was subsequently e-beam-treated and oxidatively stabilized. The resulting structure was extensively characterized by using appropriate high-resolution analytical techniques to gain a better understanding of the morphological and intrinsic properties. The achieved surface area was initially 39.95 m^2^/g and decreased to 0.99 m^2^/g for the porous CF, with the density of the latter being significantly lower than that of standard CF at 1.537 g/cm^3^. The cyclization index was determined to be 54%, indicating that the main cyclization took place in the conventional oxidative stabilization step, as opposed to cyclization and cross-linking by the e-beam. A core–sheath structure could be observed, and defects, pores, and voids could be described and demonstrated at different scales using different imaging techniques such as SEM cross-sectional images, FIB, AFM cross-sectional images, and TEM sections. WAXS showed a decrease in crystallinity from the porous precursor to the porous carbon fiber. However, for the e-beam-treated and -stabilized fibers, the largest pores could be identified using TEM measurements. These results provide a framework for a deeper understanding of the structure–property relationships along the carbon fiber production chain in order to develop tailor-made porous CF for CF-based supercapacitors.

## Notes

I.K.: Conceptualization, Methodology, Formal analysis, Writing—Original Draft, Writing—Review and Editing, Supervision, Project administration, Funding acquisition; J.W.: Formal analysis, Investigation, Writing—Original Draft; P.B.: Resources, Writing—Review and Editing, Project administration; M.S.B.: Formal analysis, Investigation, Writing—Original Draft; M.T.M.: Investigation; M.E.: Investigation; A.D.: Investigation; A.M.: Investigation; P.S.: Investigation, Visualization; F.H.: Formal analysis, Investigation; M.F.: Formal analysis, Investigation; S.J.H.: Formal analysis, Investigation, Writing—Original Draft; B.-C.K.: Resources; J.Z.: Formal analysis, Investigation, Writing—Original Draft; G.P.: Formal analysis, Investigation; G.F.: Resources; R.S.-G.: Investigation; I.K.: Resources, Writing—Review and Editing; T.B.: Resources, Visualization, Funding acquisition; P.A.L.: Resources; M.L.: Writing—Review and Editing; D.P.: Writing—Review and Editing; B.R.: Resources, Writing—Review and Editing; M.G.: Resources, Writing—Review and Editing, Funding acquisition; C.C.: Conceptualization, Resources, Writing—Review and Editing, Supervision, Funding acquisition
